# Anæsthesia, or the Employment of Chloroform and Ether in Surgery, Midwifery, &c.

**Published:** 1849-04

**Authors:** 


*Anæsthesia, or the Employment of Chloroform and Ether in Surgery, Midwifery, etc. etc.* By J. Y. SIMPSON, M. D., F. R. S. E., Professor of Midwifery in the University of Edinburg, etc. etc. Philadelphia: LINDSAY & BLAKISTON, 1849, pp. 248, 8vo.

The introduction of anæsthetic agents into medicine is among the most remarkable of the many improvements which so wonderfully characterize the present age. After full and careful experiments, their value is now abundantly demonstrated, and their place in the list of medicinal agents satisfactorily ascertained. To Professor Simpson belongs the honor of having introduced chloroform to the profession, as a substitute for ether, and of proving, by bold and persevering trials, the availability of it in moderating, and even preventing altogether, the pangs of parturition. Long doubtful of the safety of this practice, we are now convinced of its innocence and propriety; and we cannot express our feelings of gratitude to all who have in any way contributed to the procurement of this god-like boon to women. Men of tender consciences may now get married with impunity—and anticipate the happiness of paternity without the intervention of the fearful vision of agonizing labor. To men, the gift is hardly less precious than to women. Who that has seen a surgeon's saw or keen-edged knife, but has suffered in every nerve with horrible anticipations of becoming one day or other, a reluctant subject of these terrible engines of torture; and how calmly now can one lie down to sleep with the pleasant consciousness, that pain is a mere humbug; an obsolete idea; a thing of history, not of anticipation.

We are sorry that the peculiarities of the case do not permit any very general use of anæsthesia in dental operations; but the subject is yet in its infancy, and we do not know what may yet be accomplished.

This work of Prof. Simpson comprises his many papers upon the subject, and the result of his observation and information up to the time of publication, and certainly affords the most complete history of anæsthetic agents, their discovery, history and mode of application which has yet appeared. We commend it to our readers as a text-book upon this very interesting subject.

